# Kick back and destroy the ride: Alcohol-related violence and associations with drinking patterns and delinquency in adolescence

**DOI:** 10.1186/1747-597X-2-18

**Published:** 2007-07-02

**Authors:** Martin Stafström

**Affiliations:** 1 Department of Health Sciences, Division of social medicine and global health, Lund University, Sweden

## Abstract

**Aim:**

To assess how drinking patterns and delinquency are associated with self-reported experiences of alcohol-related violence in an adolescent population.

**Population and research design:**

Cross-sectional data were acquired from the Scania drug use survey 2005, consisting of 3847 students in 9^th ^grade. Abstainers were omitted and 1873 responses analyzed, with binary and multi-variable logistic regression modeling.

**Results:**

All drinking pattern indicators were statistically significantly associated with alcohol-related violence, high usual volume of distilled spirits consumed (OR 2.2, CI 95 % 1.7–2.9) being the strongest. Delinquency had, when included in the analysis, a significant effect (OR 2.5, CI 95 % 1.8–3.6); however, the drinking pattern indicators also remained statistically significant. An analysis of the effect moderation between usual volume of distilled spirits consumed and delinquency showed that there was a synergetic effect between them (SI 1.6, CI 95 % 1.1–2.4). A separate analysis for non-delinquent students, those with little experience of delinquency, and those who engaged regularly in delinquent activities, showed that the effects of different drinking patterns, especially use of distilled spirits, were significant in both groups, however, differently distributed.

**Conclusion:**

The results show that alcohol consumption pattern, with usual volume of distilled spirits being the most prominent one, had an effect on alcohol-related violence, and that this effect was amplified by delinquent behavior. The analyses also showed that there are similarities, regarding risk factors for alcohol-related violence, between delinquent and non-delinquent youth. This, indicating that consumption pattern cannot be discarded as a key factor in alcohol-related violence in adolescence.

**Policy implications:**

The study shows that alcohol-related violence in adolescence is related to both alcohol consumption patterns, e.g. usual volume of distilled spirits consumed, and delinquency. In order to prevent the harm outcome, both phenomenons have to be targeted, either by alcohol or broader social policy initiatives.

## Background

Alcohol-related violence is a serious and too common factor leading to mortality in adolescence. In the adult population, alcohol is estimated to represent 41% of male and 32% of female DALYs (Disability-Adjusted Life Years) lost through homicide [1]. These figures are even higher in countries where the drinking culture is characterized by acute intoxication, such as the Swedish youth culture [2,3]. In non-fatal injuries, alcohol is even more prevalent. In a Norwegian study, 53% of the assault victims in an emergency department assessed that the perpetrator had been intoxicated [4]. In the Netherlands, a study found that more than a third of the assault victims who attended emergency care were intoxicated when becoming victimized [5]. The results were not different in adolescent populations [6,7].

Studies investigating the association between alcohol consumption and violence have focused on violence in general [8-10]. These studies, however, have not been able to research how different drinking patterns have had an impact on alcohol-related violence. In addition, alcohol has personality altering effects, which may act as a confounder when analyzing the relationship between drinking and violence. This suggests that individuals engaging in violence when sober may have different psychological characteristics than those who become involved in fights when intoxicated. In a recent study by Swahn & Donovan [11], using longitudinal data, an association was found between alcohol consumption in general and alcohol-related violence. However, questions remain regarding different drinking patterns and their link to alcohol-related violence in adolescence.

### Theoretical perspectives

Alcohol-related violence seems to be associated with both the frequency of binge drinking [12,13] and a high general alcohol intake [14,15]. At the same time, epidemiological and cultural studies show that there is a difference in the detrimental effects of alcohol consumption, in which alcohol-related violence is one key factor [16-18].

Social and individual factors also play an important role, which is explained by different theories, i.e. Bandura's [19] social learning theory, postulating that behavior is learned through interaction with a social environment, and Jessor & Jessor [20] and their problem behavior theory, how delinquencies often are connected. Adolescents practicing one level of deviance tend to interact with peers that are at the same level of delinquency.

In Rossow et al. [8], it was reported that when controlling for criminal activity, the effect of intoxication on violent behavior was significantly reduced among Norwegian adolescents. However, these results referred to violent behavior in general, not necessarily relating it to intoxication. In order to understand whether these findings remain when only alcohol-related violence is investigated, further analysis would be needed.

Most studies that have focused on alcohol and violence have used general alcohol consumption indicators, i.e. frequency of binge drinking, average yearly consumption, or sales of alcohol, as proxies for alcohol consumption. However, as more recent research have concluded (e.g. [17]), drinking pattern has a great impact on the detrimental effect of alcohol consumption. Therefore, to understand the impact of different drinking patterns on alcohol-related violence, several alcohol consumption indicators would have to be included in an analysis.

Theories as well as prior empirical studies have shown that adolescents seem to cluster themselves. This process is based, not only, on culture and values, but also on choice of lifestyle. Rossow et al. [8] concluded that there is a significant discrepancy between deviant and non-deviant adolescents in terms of experience of violence. To expand these results further, it would be interesting to investigate the risk factors for each group independently.

**Figure 1:**
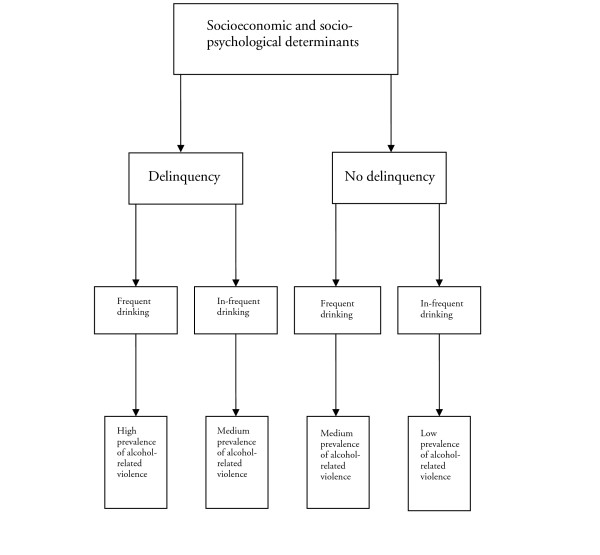
A schematic model of the associations of socioeconomic and socio-psychological determinants and drinking patterns on alcohol-related violence in adolescence.

As described, two different relationships are discussed in the literature. One is considering the impact of drinking patterns on violent behavior; the other debates the role of socio-psychology and socioeconomics. These lines of thought have been reported in several studies (i.e., [8,21]). In Figure 1, we have modeled a hypothesis of how these different theoretical assumptions may be related to each other. As illustrated, we assume that there are structural factors that have an impact on how adolescents make life decisions. Consumption patterns, however, should not be neglected as a catalyst of violence. Thus, it is likely that alcohol-related violence occurs in adolescent groups where violence otherwise is not an accepted behavior.

The aim of this study is to analyze how different aspects of drinking patterns influence the outcome of alcohol-related violence. To put these findings into context we will adjust these findings with both experiences of delinquency, but also with sensation seeking behavior, as a psychological determinant, and parental support and control to investigate the role of social learning. In addition, we will also include socioeconomic determinants, based on parents' educational level and individual financial capacity.

## Method

The current survey was conducted in the end of February 2005 among 9^th ^grade students in 16 municipalities in the county of Scania in Southern Sweden. All schools and students in the municipalities were included in the survey population, apart from 12 classes that had been selected to participate in a nation-wide survey. The questionnaires were mailed to the schools and then mailed back for data processing. All students who were present on the day of the study were asked to complete the questionnaire. The overall response rate was 86.8%. Some questionnaires contained inconsistent information, e.g. checking all of the answer alternatives or highly exaggerated alcohol consumption. These were deleted from the data, resulting in internal non-response. In total, 3847 questionnaires were available for analysis. We then omitted the respondents who reported that they had never had any experience of alcohol intoxication and the questionnaires with a non-response on the outcome variable. In total, we analyzed 1873 responses.

The questionnaires were completed anonymously. All items were based on a national Swedish school survey on tobacco, alcohol, and drug use among 9^th ^graders. The questionnaire contained 60 items and included space for comments.

### Dependent variable

Alcohol-related violence: Respondents were asked: "Have you ever been involved in a fight, as a result of alcohol intoxication". The answer alternatives were: (1) Never, (2) once, (3) twice, or (4) three or more times. This variable was then dichotomized; the students who reported one or more experiences of alcohol-related violence were coded into this category. Other items regarding alcohol-related problems included in the questionnaire were; having an argument, lost money or other valuables, destroyed clothes or other belongings, have had unwanted sex, and have had unprotected sex.

### Independent variables

Frequency of binge drinking: This item was based on the following question: "How often do you drink 1/4 of a bottle of distilled spirits (185 ml), or a bottle of wine, or four cans of full strength beer, or four cans of full strength cider/alco-pops or six cans of medium strength beer?" The answer alternatives were: (1) Never, (2) once a year or less often, (3) a few times per year, (4) once a month, (5) couple of times per month, and (6) at least once a week. Respondents reporting answer alternatives 4 to 6 were coded to be frequent binge drinkers.

#### Frequency of drinking distilled spirits/full strength beer

The variable was based on the following questions: "Usually, how often do you drink distilled spirits (either straight or mixed into a long-drink or cocktail)/full strength beer? The answer alternatives were: (1) Never, (2) once a year or less often, (3) 2–6 times per year, (4) once a month, (5) twice per month, (6) once a week, (7) twice a week, (8) every second day, and (9) every day. Students that checked alternatives 4–9 were coded as being frequent consumers of each beverage.

#### Usual volume of distilled spirits/full strength beer

The variable was based calculated from the following questions: "Usually, how much do you drink distilled spirits (either straight or mixed into a long-drink or cocktail)/full strength beer? 10 answer alternatives were given for each beverage, apart from answering that one did not consume it at all. The 10 alternatives ranged, for beer, from 330 ml or less to 4 liters or more. For distilled spirits, the answer alternatives ranged from 20 ml to 750 ml or more. Respondents indicating a consumption level exceeding 150 cl of full strength beer were coded as consuming a high usual volume for that beverage, while those students with a usual consumption level exceeding 25 cl of distilled spirits were coded as having a high usual volume for that beverage.

#### Sensation seeking behavior

The BSSS-4 index [23] was used as the indicator for sensation seeking behavior. In our sample, we obtained a Cronbach's alpha value of 0.75 for the four items, which were: (4) I would like to explore strange places, (2) I like to experience frightening things, (3) I like new and exciting experiences; even if I have to break the rules, (4) I prefer friends who are exciting and unpredictable. The answer alternatives were graded on a 5-point scale, ranging from strongly disagrees to strongly agree. The four questions were added together, making the lowest score of sensation seeking behavior 4, and the highest 20.

#### Parental control and support

In the questionnaire, we had included 8 items from the Parents control scale [24]. These were organized into a scale, which, according to a reliability analysis, had a Cronbach's alpha of 0.71. The students were asked how frequently eight different situations took place in their home. The answer alternatives were: (1) never, (2) rarely, (3) sometimes, (4) often, and (5) always. The eight situations were: (1) Do you need your parents' permission to be out late on a weeknight? (2) Do you need your parents' permission to be out late on a Saturday night? (3) If you go out on a Saturday night, do you have to tell your parents where you are going to go, and whom you are going to meet? (4) Do you tell your parents about things that you have experienced, even if it makes you feel ashamed or embarrassed? (5) Do your parents let you participate in the family's decision making process? (6) Are you interrupted when you are arguing at home? (7) Does everyone in the family eat dinner together? (8) Does it feel like your parents have confidence in you, and let you take your own responsibilities? For each answer the student could get a score from 1 (never) to 5 (always). The eight questions were added together, making the lowest score of parental control 8, and the highest 40.

#### Delinquency last year

Respondents were asked how frequent in the 12 months prior to the survey they had been personally involved a number of problem behaviors, e.g. substance use (illicit drugs and solvents), repeated use of cannabis, truancy, shoplifting, theft, robbery, and burglary. The variable was then constructed by summing the number of items in which the respondents had answered to be active in [24].

#### Parents' level of education

The questionnaire asked the students on what the highest level of education their parents, mother and father respectively, had obtained. The answer alternatives were: (1) University degree, (2) High school diploma, (3) Only primary school. The two variables – mother and father – were added together, obtaining this variable, with a range from 2 to 6, where '2' indicated the highest level of education and '6' the lowest. It should be noted that Sweden is among the most gender equal countries in the world, why using only one of the parent's educational level would cause analytical problems.

#### Individual purchasing power

The students were asked, "How much spending money do you have every month?" The answer alternatives were: (1) Less than 200 SEK, (2) 200–499 SEK, (3) 500–799 SEK, (4) 800–1099 SEK, (5) 1100–1399 SEK, (6) 1400–1699 SEK, (7) 1700–1999 SEK, and (8) 2000 SEK or more (100 SEK = 11 • or 16 US$).

In addition to these to variables, sex was also included as an independent variable, throughout the analyses.

### Statistical methods

Logistic regression modeling was performed to obtain multivariate analyses to assess the adjusted associations between independent and dependent variables. In addition, a synergy index (SI) was calculated to disclose effect modification between the chosen variables. The following algorithm was used, whereby SI > 1 signifies a synergistic effect (representing a positive effect modification) and SI < 1 an antagonistic effect (representing a negative effect modification) [25]:

SI=(OR(1+1)−1)(OR(1+0)−1)+(OR0+1)−1)
 MathType@MTEF@5@5@+=feaafiart1ev1aaatCvAUfKttLearuWrP9MDH5MBPbIqV92AaeXatLxBI9gBaebbnrfifHhDYfgasaacH8akY=wiFfYdH8Gipec8Eeeu0xXdbba9frFj0=OqFfea0dXdd9vqai=hGuQ8kuc9pgc9s8qqaq=dirpe0xb9q8qiLsFr0=vr0=vr0dc8meaabaqaciaacaGaaeqabaqabeGadaaakeaacqqGtbWucqqGjbqscqGH9aqpdaWcaaqaaiabcIcaOiabb+eapjabbkfasnaaBaaaleaacqGGOaakcqaIXaqmcqGHRaWkcqaIXaqmcqGGPaqkaeqaaOGaeyOeI0IaeGymaeJaeiykaKcabaGaeiikaGIaee4ta8KaeeOuai1aaSbaaSqaaiabcIcaOiabigdaXiabgUcaRiabicdaWiabcMcaPaqabaGccqGHsislcqaIXaqmcqGGPaqkcqGHRaWkcqGGOaakcqqGpbWtcqqGsbGudaWgaaWcbaGaeGimaaJaey4kaSIaeGymaeJaeiykaKcabeaakiabgkHiTiabigdaXiabcMcaPaaaaaa@4FA9@

where: OR_(1+1) _= odds ratio for dummy variable exposed to both factors

OR_(1+0) _= odds ratio for dummy variable exposed to one factor

OR_(0+1) _= odds ratio for dummy variable exposed to other factor

OR_(0+0) _= odds ratio for the dummy variable unexposed to both factors

Significant effect moderation between two variables indicates that when both are present there is an amplified effect, i.e., that the combination of the two indicators has a stronger effect, which is higher than added effect of the variables in question.

## Results

Among the 1873 analyzed questionnaires, 567 (30.3%) respondents reported to have experienced alcohol-related violence. Of these, 265 had one such experience, 110 had two, while 192 had three or more. Table 1 shows each independent variable in relation to alcohol-related violence. The tendency was that the number of experiences of alcohol-related violence increased with alcohol consumption. This was also true for delinquency. In the sample, the majority of students had not binged, nor had they consumed smaller volumes on a monthly basis. In addition, most respondents usually consumed rather small quantities of alcohol when they drank.

**Table 1:** Descriptive statistics of the variables included in the analyses

	Alcohol-related violence							
	Never		1 time		2 times		3 or more times	
	N	%	N	%	N	%	N	%
Gender								
- Boys	612	64.8	141	14.9	68	7.2	123	13.0
- Girls	692	74.9	124	13.4	41	4.4	67	7.3
Frequency of binge drinking								
Never	164	90.6	12	6.6	0	0.0	5	2.8
- Once a year or less often	223	85.4	22	8.4	7	2.7	9	3.4
- A few times per year	343	78.3	50	11.4	15	3.4	30	6.8
- Once a month	340	67.9	83	16.6	33	6.6	45	9.0
- Couple of times per month	159	50.5	66	21.0	31	9.8	59	18.7
- At least once a week	46	33.3	29	21.0	20	14.5	43	31.2
Frequency of drinking full strength beer								
- Less than once a month	742	79.9	101	10.9	33	3.6	53	5.7
- Once a month ore more often	564	59.7	164	17.4	77	8.2	139	14.7
Frequency of drinking distilled spirits								
- Less than once a month	666	83.1	79	9.9	17	2.1	39	4.9
- Once a month ore more often	640	59.7	186	17.4	93	8.7	153	14.3
Usual volume of full strength beer								
- 2 cans or less (1000 ml)	726	78.3	108	11.7	34	3.7	59	6.4
- 3 cans or more	371	56.5	115	17.5	61	9.3	110	16.7
Usual volume of distilled spirits								
- 3 shots (120 ml) or less	756	81.9	98	10.6	27	2.9	42	4.6
- More than 3 shots	496	56.8	156	17.9	76	8.7	145	16.6
Delinquency the last 12 months								
- No	466	85.7	52	9.6	13	2.4	13	2.4
- Yes	787	64.6	194	15.9	86	7.1	151	12.4

We modeled three different logistic regressions in order to investigate the relationship between the independent variables and the dependent (Table 2). In Model 1, we analyzed the relationship between alcohol drinking patterns and alcohol-related violence, disregarding other confounders. As shown, there were significant associations between all the alcohol consumption indicators and alcohol-related violence. In Model 2, we included sensation seeking behavior, parental control and support, parents' education level, and individual purchasing power into the analysis, which did not change the impact of the drinking behavior. In Model 3, delinquency was included. As seen, all the alcohol consumption pattern indicators remained significant. However, the delinquency variable showed to be highly significant.

**Table 2:** Results from logistic regressions of alcohol-related violence on drinking patterns (Model 1), then adding parents' education level, parental control and support, sensations seeking behavior, and purchasing power (Model 2), and, finally, including delinquency the last year (Model 3)

**MODEL I**		OR	95% CI	Wald test
	Sex^a^	1.0	0.80 – 1.35	0.079
	Frequent binge drinking	1.4	1.03 – 1.93	4.580
	Drinking full strength beer twice a month or more often	1.5	1.06 – 2.01	5.392
	Drinking distilled spirits twice a month or more often	1.9	1.38 – 2.60	15.874
	Usual volume of full strength beer exceeds 150 cl	1.4	1.05 – 1.86	5.377
	Usual volume of distilled spirits exceeds 25 cl	2.2	1.73 – 2.92	36.960

**MODEL II**	Sex^a^	0.9	0.68 – 1.19	0.593
	Frequent binge drinking	1.4	1.01 – 1.97	4.082
	Drinking full strength beer twice a month or more often	1.4	1.02 – 2.01	4.246
	Drinking distilled spirits twice a month or more often	1.8	1.28 – 2.47	11.513
	Usual volume of full strength beer exceeds 150 cl	1.5	1.14 – 2.09	7.813
	Usual volume of distilled spirits exceeds 25 cl	2.1	1.56 – 2.72	26.044

**MODEL III**	Sex^a^	0.9	0.69 – 1.24	0.298
	Frequent binge drinking	1.5	1.05 – 2.09	4.977
	Drinking full strength beer twice a month or more often	1.4	0.98 – 2.00	3.485
	Drinking distilled spirits twice a month or more often	1.7	1.16 – 2.35	7.798
	Usual volume of full strength beer exceeds 150 cl	1.6	1.14 – 2.14	7.797
	Usual volume of distilled spirits exceeds 25 cl	1.9	1.40 – 2.51	17.852
	Delinquency in the last year	2.5	1.78 – 3.57	26.036

df = 1

^a ^Reference category = Girls

This far in the analysis it was clear that there was a very strong association between delinquent behavior and alcohol-related violence. In order to deepen the analysis, the sample was divided into three different groups based on delinquency in the last year (Table 3), no such experience, 1–2 experiences, and more than 3 such experiences.

**Table 3:** Effect moderation of alcohol-related violence between frequent binge drinking and delinquency, expressed as Synergy Index (SI)

*Variables*	N	OR	CI (95%)	Wald test	SI	CI (95%)
Non-delinquent and low usual consumption of distilled spirits	440	1.00		154.778		
Delinquent and low usual consumption of distilled spirits	861	2.3	2.35 – 4.94	41.764		
Non-delinquent and high usual consumption of distilled spirits	150	2.4	2.36 – 6.46	28.118		
Delinquent and high usual consumption of distilled spirits	519	9.7	6.62 – 14.22	135.742	1.64	1.13–2.37

The strong association between delinquency and alcohol-related violence, which was anticipated, led us to conduct an analysis of the effect moderation between usual volume of distilled spirits consumed and delinquency (Table 3). In this, we created a dummy variable consisting of four different categories as seen in the table, and we adjusted the logistic regression for sex. The reference category in this analysis was to have a relatively low usual volume of distilled spirits consumed and no experience of delinquency in the last 12 months. The odds ratios for having one of the other were significantly associated with alcohol-related violence. In addition, the relationship between alcohol-related violence and the final category, i.e. to have a relatively high usual volume of distilled spirits consumed and to have had experience delinquency in the last year, were statistically stronger than the mere additive one. Thereby, the results showed that there was a synergetic effect, since there was a statistically significant interaction between the two dependent variables, which implies that they have an amplifying effect on the outcome.

In order to more thoroughly investigate the relationship between delinquency and alcohol-related violence, while taking consumption pattern into consideration, we divided the sample into three groups; non-delinquents, those who had had one or two experiences of delinquency, and finally, those students who had engaged in delinquent activities three or more times in the last year. As shown in Table 4, among the students not being involved in delinquency in the last year (Model 1), only high usual volume of distilled spirits consumed was significantly associated with alcohol-related violence. High usual volume of full strength beer also seems to be correlated with the outcome variable, however, though not statistically significant. Among the students who had been involved in delinquency one or two times in the last year (Model 2), we only frequent use of distilled spirits statistically significant associated with the outcome variable. In the group of adolescents with more than three experiences of delinquency in the last year, it was yet again high usual consumed volumes that were statistically significant determinants for alcohol-related violence.

**Table 4:** Comparison of students with no experience of delinquency in the last 12 months (n = 544) (Model 1), and those with one or two such experiences (n = 805) (Model 2), and finally, those with three or more delinquent experiences (n = 413) (Model 3).

**MODEL I**		OR	95% CI	Wald test
	Sex^a^	1.3	0.59 – 2.73	0.370
	Frequent binge drinking	1.3	0.58 – 2.85	0.372
	Drinking full strength beer twice a month or more often	1.0	0.41 – 2.65	0.006
	Drinking distilled spirits twice a month or more often	1.2	0.52 – 3.01	0.236
	Usual volume of full strength beer exceeds 150 cl	2.2	1.00 – 4.90	3.835
	Usual volume of distilled spirits exceeds 25 cl	3.0	1.52 – 5.73	10.270

**MODEL II**	Sex^a^	0.8	0.54 – 1.31	0.564
	Frequent binge drinking	1.4	0.86 – 2.28	1.576
	Drinking full strength beer twice a month or more often	1.4	0.89 – 2.43	2.034
	Drinking distilled spirits twice a month or more often	2.6	1.58 – 4.35	13.231
	Usual volume of full strength beer exceeds 150 cl	1.2	0.77 – 1.86	0.563
	Usual volume of distilled spirits exceeds 25 cl	1.4	0.89 – 2.09	2.381

**MODEL III**	Sex^a^	0.9	0.53 – 1.46	0.239
	Frequent binge drinking	1.5	0.75 – 2.85	1.266
	Drinking full strength beer twice a month or more often	1.7	0.90 – 3.17	2.632
	Drinking distilled spirits twice a month or more often	1.1	0.58 – 2.09	0.094
	Usual volume of full strength beer exceeds 150 cl	1.7	0.98 – 3.01	3.585
	Usual volume of distilled spirits exceeds 25 cl	1.9	1.13 – 3.30	5.771

df = 1

^a ^Reference category = Girls

Finally, we conducted an analysis of the effect moderation between usual volume of distilled spirits consumed and delinquency (Table 3). In this analysis we created a dummy variable consisting of four different categories as seen in the table, and we adjusted the logistic regression for sex. The reference category in this analysis was to have a relatively low usual volume of distilled spirits consumed and no experience of delinquency in the last 12 months. The odds ratios for having one of the other were significantly associated with alcohol-related violence. In addition, the relationship between alcohol-related violence and the final category, i.e. to have a relatively high usual volume of distilled spirits consumed and to have had experience delinquency in the last year, were statistically stronger than the mere additive one. Thereby, the results showed that there was a synergetic effect, which implies that the two independent variables combined have an amplifying effect on the outcome.

## Discussion

As shown by others [8,22], there is an association between alcohol consumption and violent behavior in adolescence. In order to understand this association we hypothesized the associations in Figure 1. As our analyses have shown the model described earlier seems to be valid in describing the relationship between drinking patterns, delinquency in general, and alcohol-related violence. The logistic regression models in this paper showed that there were strong simultaneous associations between drinking pattern and delinquency with alcohol-related violence. The analysis of the effect moderation between usual volume of distilled spirits and delinquency showed that there was a synergetic effect between binge drinking and delinquency. These findings strongly suggest that alcohol-related violence cannot be explained by delinquency or drinking single-handedly, as both behaviors contributed to the outcome.

Different drinking patterns were shown to have impact on alcohol-related violence, and among the consumption of distilled spirits was the most prominent one. This finding supports the assumption that the degree of alcoholic intoxication is a risk factor in itself for alcohol-related violence. The results, in addition, contradict the hypothesis brought forward by White et al. [21] that aggressive behavior in general is a prerequisite for alcohol-related violence. These findings show that, likely, alcohol consumption per se brings on, otherwise latent, aggression. This process is enforced when a person at risk consumes beverages of higher potency (i.e. distilled spirits) or if the consumption is frequent. Among the students that reported to have participated in delinquency and the ones reporting no such experiences the usual volume of distilled spirits was the indicator with the strongest association to alcohol-related violence. However, among the students with on to two delinquent experiences, it was rather the frequency of drinking that seemed to be more important. Further analysis would be needed to give this discrepancy a deeper understanding, however, as the results showed, the type of beverage remains important.

That the results showed beverage specific discrepancies did not come as surprise. The stronger the drink, the more planning and care is needed to not become exaggeratedly intoxicated. Among adolescents, the number of drinking experiences is still limited, which may explain this difference between beverages. On the other hand, it is also important input for policy-makers. A measure to reduce harm would, therefore, be to limit the marketing possibilities of liquor brands and products to the population at focus in this study.

Another interesting finding of this study is the similarities between different sub-groups. The risk factors for alcohol-related violence were similar among the non-delinquents and the more regular ones. It is likely that the level of interaction between these two groups is rather limited, based on the research by Jessor & Jessor [20]. A similarity, however, is the potency of distilled spirits and the detrimental effects of intoxication. This indicates that consumption pattern cannot be discarded as a key factor in alcohol-related violence in adolescence.

### Methodological considerations

The methodology of collecting cross-sectional data regarding alcohol and drug consumption behavior and the validity of this methodology has been discussed in several studies. The results have consistently shown school surveys on alcohol and drug use to be valid. [19,20]

Even so, it is important to be aware of the potential pitfalls. There is a risk of dependent misclassification. For example, there may be students whose parents disapprove of them drinking. As a result, these students may be reluctant to report the fact that they consume large quantities of alcohol. However, since the survey was conducted anonymously, the risk of this kind of misclassification should be reduced. Selection bias is another methodological trait. In this case the municipalities conducted a sampling of all 9^th ^grade students within the public school system, and since more than 95% of the 9^th ^graders are within this system in the municipalities in question selection bias should not have constituted a problem.

In the analysis, we used stratifications of the delinquency variable (Table 4). This approach may have resulted in within stratum confounding. However, the stratification was used to make the results more interpretable, and the risk of residual confounding should not overshadow that objective.

We have found no evidence of confounding in the main exposure variables. However, the questionnaire item that was used to record alcohol-related violence could be interpreted differently by individual respondents, leading to non-differential misclassification. This also applies for the selection criteria – to have been intoxicated. The latter one, should constitute a smaller problem, since if one considers oneself to never have been intoxicated, it should also be difficult to consider one to have encountered problems due to ones own alcohol consumption.

## Conclusion

The findings in this study suggest that alcohol consumption patterns, and then especially usual volume consumed, are as important as delinquency when disentangling alcohol-related violence among adolescents. In addition, the results also indicate that delinquency and high usual volume of distilled spirits consumed amplifies, by a synergetic relationship, the effect on alcohol-related violence. Finally, this study clearly shows that the similar drinking patterns have impact on alcohol-related violence in adolescence, no matter the degree of delinquency. This can be seen as an indication that consumption pattern cannot be discarded as a key factor in alcohol-related violence in adolescence.

## Policy implications

Overall, these results indicate that alcohol-related violence cannot be prevented unless there is both a reduction in delinquency and alcohol use in adolescence. Harm is also related to the type of alcoholic beverage. Therefore, these results would strongly suggest that distilled spirits should not be marketed towards younger people. Such limitations should include both products as brands. Another preventive measure would be to implement policies that affect the delinquency levels in the adolescent population.

## Competing interests

The author(s) declare that they have no competing interests.
